# The Global Prevalence of Schizophrenia

**DOI:** 10.1371/journal.pmed.0020151

**Published:** 2005-05-31

**Authors:** Dinesh Bhugra

## Abstract

Bhugra discusses the implications of a new study in *PLoS Medicine* that challenges widely held assumptions about the epidemiology of schizophrenia.

There is no doubt that the global burden of schizophrenia, a chronic serious mental illness, is massive. It is therefore essential that any intervention is appropriate, cost-effective, and efficacious.

To reduce the burden, a clear epidemiologically based dataset is required. The first step in such a venture is provided by Saha et al. in their systematic review of prevalence data on schizophrenia across cultures, published in this month's *PLoS Medicine* [1]. Using a number of strategies these authors have distilled the findings from just under 200 studies from 46 nations.

## Defining the Prevalence of the Disease

The prevalence rates of schizophrenia depend upon a whole range of factors, such as the availability of and response to treatment. The prevalence of schizophrenia, as with other mental disorders, can be calculated from a number of sources—from case registers to field surveys. The latter lend themselves more readily to estimation of period prevalence (see Glossary) than point prevalence, while case register data can provide point prevalence more readily. The denominator can be the whole population or only a small defined population.

Saha et al. quite rightly differentiate between traditional prevalence, or “core”, studies (these generate an estimate based on the population residing within a defined catchment area), and studies in specific sub-groups (which they divide into migrant studies and studies in other special groups). Using sequential filters they were able to isolate discrete data from multiple studies, and they used other strategies to ensure that the largest groups were counted.

The authors had hypothesised that prevalence estimates would differ between lifetime, period, and point prevalence and that lifetime prevalence would be higher than lifetime estimates (estimates are calculated as a proportion by dividing the total number of individuals who manifest a disorder [the numerator] by the total population at risk including those with the disorder [the denominator]). They also predicted that the estimates would be higher for males, those from urban areas, and migrants. Case ascertainment methods and sample selection do influence prevalence rates, so Saha et al. chose studies that used comprehensive case ascertainment methods.

## Findings of the Study

Of the 132 core studies, 21 studies reported point prevalence, 34 reported period prevalence, and 24 reported lifetime prevalence. The median prevalence of schizophrenia was 4.6/1,000 for point prevalence, 3.3/1,000 for period prevalence, 4.0 for lifetime prevalence, and 7.2 for lifetime morbid risk.

There were no significant differences between males and females, nor between urban, rural, and mixed sites, although migrants and homeless people had higher rates of schizophrenia and, not surprisingly, developing countries had lower prevalence rates (the lower prevalence of schizophrenia in developing countries has been previously documented). It is well known from other studies that migrants have higher than expected rates of schizophrenia [2–8], although definitions of migrants in these studies have been variable and the studies have suffered from a series of other methodological problems.

## Implications of the Study

Several important findings emerge from Saha and colleagues' analysis. For clinicians, the analysis indicates clearly that lifetime prevalence is 4.0/1,000 and not 1%, as reported in the *Diagnostic and Statistic Manual of Mental Disorders*, fourth edition [9], and other textbooks.

The study also challenges the widely held view that schizophrenia is much more common in men. Saha et al.'s finding that schizophrenia was just as common in women has clear implications for developing services, since it means that not only must we develop and provide culturally appropriate services but also services that are gender sensitive (as the number of cases in women are higher than expected, gender becomes a more important factor). Furthermore, if the prodrome of the illness is long, this will affect the number of new cases appearing in the population, and longer delays in treatment will also affect the rates.[Fig pmed-0020151-g001]

**Figure pmed-0020151-g001:**
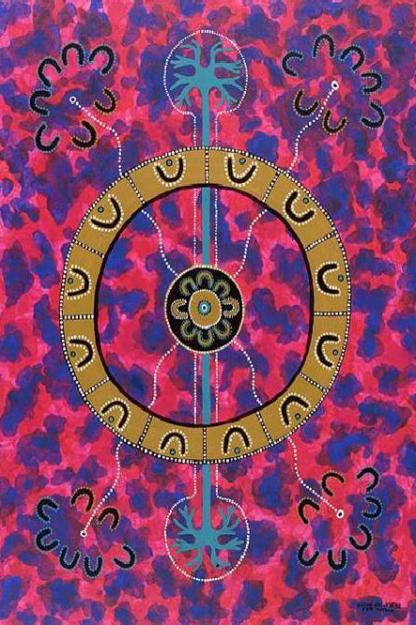
Psychiatric Research by Ted Watson—this painting, representing collaborative research between people with schizophrenia and mental health professionals, is by an aboriginal mental health service user and was commissioned by the Queensland Centre for Mental Health Research, Australia

An important question for the researchers is whether cases counted as positive included patients with positive and/or negative symptoms, those who may have had underlying cognitive deficits, and those whose illness was treatment resistant.

Detailed economic measures must be included in analyses of the prevalence of schizophrenia to determine whether countries are developed or developing. Saha et al. themselves acknowledge that they have used a single measure of World Bank definitions relying on per capita income (whereas in any country there will be marked geographical variation in social and economic classes) for assessing a complex and multi-dimensional concept, which is a weak point of their systematic review. The impact of urbanisation must be studied especially, as in many developing or low-income countries the migration into urban areas adds a tremendous amount of variation that must be taken into account in future ecological studies.

The authors acknowledge that their systematic review may have missed some studies, and they encourage readers who know about missing studies to contact them.

## Schizophrenia across Cultures

Studies have shown that the outcome of schizophrenia is better in developing countries [10–12], and therefore the point prevalence in these countries should be lower. Despite this clear difference in the course of schizophrenia in different cultures, cross-cultural research in psychiatry focuses on similarities rather than differences. Kleinman suggests that there is a very strong bias towards discovering universals in mental disorder [13]. Both the International Pilot Study of Schizophrenia [10] and the Determinants of Outcome of Severe Mental Disorders study [12] used a template of symptoms of psychosis across cultures to identify groups of patients who seemed similar, but these studies left out all those patients who failed to fit the template. It is these excluded patients that Kleinman suggests are of greater interest from a cultural perspective simply because they are the ones who would reveal the greatest amount of cultural diversity.

In the International Pilot Study of Schizophrenia [10] and the Determinants of Outcome of Severe Mental Disorders study [12], catatonia (a form of schizophrenia characterized by a tendency to remain in a fixed stuporous state for long periods) was diagnosed in 10% of cases in developing countries compared with less than 1% in developed countries. Hebephrenia (a form of schizophrenia characterized by severe disintegration of personality) was present in 13% of cases in developed countries and 4% in developing countries. These differences in the disease in developed versus developing countries indicate that there is more to the prevalence of schizophrenia than simple epidemiological data. Better prognosis in developing countries may indicate different sets of aetiological and perpetuating factors [14].

Cohen [15] argued that although the case-finding method in both these studies was accurate, the vast majority of cases were identified in Western-type facilities, and therefore the numbers of true cases of schizophrenia may be an underestimate. He also pointed out that in developing countries the proportion of cases with acute onset schizophrenia was twice as high as in developed countries. Such variations may indicate a real difference in the cross-cultural manifestations of schizophrenia—hospital-based data collection reflects cultural processes that have little to do with the true prevalence and incidence rates of schizophrenia.

While prevalence studies can help contribute to an understanding of the aetiology of schizophrenia, psychodynamic issues—such as cultural identity and attachment—must also be studied, especially among migrant groups, as cultural congruity and ethnic density may influence the presentation of suffering individuals to psychiatric services [16,17].

**Glossary**

**Point prevalence:** The proportion of individuals who manifest a disorder at a given point in time.

**Period prevalence:** The proportion of individuals who manifest a disorder over a specific period of time (e.g., over one year).

**Lifetime prevalence:** The proportion of individuals in the population who have ever manifested a disorder, who are alive on a given day.

**Lifetime morbid risk:** The probability of a person developing the disorder during a specified period of their life or up to a specified age (lifetime morbid risk differs from lifetime prevalence in that it attempts to include the entire lifetime of a birth cohort both past and future, and includes those deceased at the time of the survey).
